# Enhancing Facial Rejuvenation Outcomes With a Novel Retinaldehyde‐Based Cream: A Comparative Randomized Intra‐Individual Study

**DOI:** 10.1111/jocd.70555

**Published:** 2025-11-28

**Authors:** Camille Monteil, Valeria Barreto‐Campos, Edward Lain, Emma Guillou, Gautier Doat, Thérèse Nocera

**Affiliations:** ^1^ Research and Development Pierre Fabre Dermo‐Cosmétique & Personal Care Toulouse France; ^2^ Visiting Professor of Dermatology and Laser at Jundiaí Medical School Sao Paulo Brazil; ^3^ Sanova Dermatology, Austin Institute for Clinical Research Austin Texas USA; ^4^ Laboratoires Dermatologiques Eau Thermale Avène, Pierre Fabre Dermo‐Cosmétique & Personal Care Lavaur France

**Keywords:** anti‐aging, controlled study, dermocosmetic, filler, rejuvenation

## Abstract

**Background:**

Minimally‐to‐moderately invasive facial rejuvenation procedures, such as chemical peels, hyaluronic acid (HA) injections and fractional lasers, yield visible improvements in skin texture, tone and wrinkles; their long‐term benefits can be optimized through a targeted anti‐aging skincare regimen.

**Aims:**

This controlled study aimed to evaluate the tolerability and efficacy of a cream containing three evidence‐based active ingredients (retinaldehyde, niacinamide and haritaki fruit extract) to support and maintain the benefits of minimally to moderately invasive facial rejuvenation procedures.

**Methods:**

A monocentric, controlled, randomized, split‐face study was conducted over a three‐month period in subjects who had undergone one type of rejuvenation procedure, with assessments beginning after re‐epidermization. Subjects were instructed to apply the test product on the randomized hemiface once daily.

**Results:**

This comparative controlled study, conducted under close dermatological supervision in 66 subjects who had undergone either chemical peels, HA injections, or fractional laser (*n* = 22 per group), demonstrated that the cream significantly improved multiple signs of aging. Throughout the entire study, compliance was very good. At 1 month (M1), wrinkles were significantly reduced, and the skin was significantly firmer and plumper, with improved texture. Skin tone homogeneity and radiance were significantly enhanced as of M1 (and M2 for skin smoothness) versus the control hemiface.

**Conclusion:**

Regular application of the study product, which contains retinaldehyde, niacinamide, and haritaki fruit extract, yielded significantly visible anti‐aging results already at 1 month compared with the control, with very good skin tolerance. It can be used to maximize and maintain the benefits of rejuvenation procedures and promote long‐term skin health.

**Trial Registration:**

ClinicalTrials.gov identifier: NCT06942403

**Abbreviations**
AA: anti‐aging
AE: adverse event
CHMP: Committee for Medicinal Products for Human Use
CO_2_
: carbon dioxide
D1, D8, M1, M2, and M3: Day 1, Day 8, Month 1, Month 2, and Month 3, referring to study time points
EMA: European Medicines Agency
FAS: full analysis set
GCP: Good Clinical Practice
HA: hyaluronic acid
HRIPT: Human Repeated Insult Patch Test
IL: interleukin
NRS: numerical rating scale
ROS: reactive oxygen species
SASP: senescence‐associated secretory phenotype
SEM: standard error of the mean

## 1 Introduction

Dermocosmetics play a significant role in the dermatologist's arsenal of treatments for use after anti‐aging procedures, helping to strengthen the skin's barrier effect and providing antioxidant and soothing properties. Minimally to moderately invasive dermatological rejuvenation procedures such as chemical peels, hyaluronic acid (HA) injections and fractional lasers, are widely used to address signs of aging and enhance skin quality thanks to their effectiveness and relatively minimal associated downtime [1, 2].

Superficial glycolic acid peels (30%–50%) exfoliate the skin, enhancing texture, tone, and radiance while reducing fine lines and pigmentation [3]. Hyaluronic acid (HA) injections restore hydration, smooth fine lines, and boost elasticity, offering both immediate plumping and long‐term rejuvenation [4]. Fractional ablative laser stimulates collagen neosynthesis, improving skin laxity, with moderate downtime [5].

Following these minimally‐to‐moderately invasive treatments, the skin undergoes repair and regeneration [6, 7].

While these procedures yield visible improvements in skin texture, tone and wrinkles, their long‐term benefits can be significantly optimized through the use of a targeted anti‐aging skincare regimen. New cosmeceutical protocols can be used as an adjunctive strategy, rendering anti‐aging regimens more efficient for patients [8, 9, 10, 11]. Their tolerance and efficacy must be assessed carefully using ingredients of proven efficacy and a well‐tolerated formulation suitable for use as part of a post‐procedure skincare regimen [12]. A number of select active ingredients have demonstrated efficiency in targeting skin aging processes, including retinal (retinaldehyde) and niacinamide [12]. Both have been incorporated in a cream containing haritaki fruit extract (a standardized extract of *Terminalia chebula* fruit) and have shown to display antioxidant, anti‐inflammatory [13] and anti‐aging properties [14].

To support and maintain the benefits of minimally‐to‐moderately invasive facial rejuvenation procedures, this study aimed to evaluate the tolerability and efficacy of a cream containing the three evidence‐based active ingredients (retinaldehyde, niacinamide and haritaki fruit extract). A monocentric, controlled, randomized, split‐face study was conducted over a three‐month period in subjects who had undergone one of three types of rejuvenation procedures, with assessment beginning after re‐epidermization.

## 2 Material and Methods

### 2.1 Study Design and Ethics

This monocentric, intra‐individually controlled, randomized, split‐face study was conducted in Poland (September 25, 2023, to March 31, 2024). For each participant, the study lasted 98 days with 5 evaluation time points: day 1 (D1, inclusion visit, anti‐aging procedure), D8, D8 + 1 month (M1), D8 + 2 months (M2), and D8 + 3 months (M3).

This study was performed in accordance with the principles set out in the Declaration of Helsinki and its subsequent amendments (1964, and the most recent amendment in force), and in accordance with the spirit of Good Clinical Practice (GCP) Guideline published by the International Conference on Harmonization (Adopted by CHMP, December 15, 2016, issued as EMA/CHMP/ICH/135/1995) and local regulatory requirements. The protocol was approved by a local ethics committee. It also conformed to Regulation (EC) No. 1223/2009 of the European Commission and French Decree No. 2017‐884 pertaining to research on cosmetic products.

As registration of studies evaluating cosmetic products is not mandatory in the European Union, the study was retrospectively registered under clinical trial (April 24, 2025) for publication purposes. Informed consent (and authorization for use of photographs) was obtained from all subjects during inclusion.

### 2.2 Study Population

Female and male volunteers (aged 35–70 years), of phototypes I–IV according to the Fitzpatrick classification, of all skin types, and considered healthy by the investigator, were included if they had planned a facial rejuvenation procedure including for crow's feet wrinkles: peeling (30% or 50% glycolic acid) for 22 subjects, fractional ablative CO_2_ laser for 22 subjects and injections of HA or a mix containing HA on the crow's foot area for 22 subjects. All procedures were to be minimally to moderately invasive and aimed at achieving an anti‐aging effect.

Pregnant or breastfeeding women, and those with dermatological or medical conditions considered incompatible with the study or potentially hazardous, including abnormal reactions to sunlight or hypersensitivity to cosmetic products, were excluded. Subjects receiving topical or systemic treatments likely to interfere with study data, those who had used self‐tanning products or experienced artificial UV exposure or excessive natural sun exposure in the month prior to inclusion, were also considered ineligible. Additional exclusion criteria included prior or planned surgery, chemical treatment, or significant invasive dermal procedures on the study areas that could interfere with study data (as judged by the investigator). The use of any skincare or makeup products other than their usual cleanser on the day of the inclusion visit was also prohibited.

During the study, subjects were required to avoid exposure to sunlight and UV rays, and the use of any concomitant treatments or interventions deemed likely to interfere with the study results was prohibited.

### 2.3 Products and Application Procedures

Following the anti‐aging procedure at D1 (inclusion visit), the subjects applied a reparative product (containing [C^+^‐Restore]) at home from D1 to D7 on the target area for subjects having received injections, and on the whole face for all other subjects at least twice a day, before receiving the test product and a hydrating cream (Avène hydrating cream) at D8.

The test product (Avène 0.1 cream) is an anti‐aging (AA) cream containing 0.1% of retinaldehyde, 2% of niacinamide and 0.1% of dry extract of haritaki fruit as the active ingredients.

Subjects were instructed to apply the test product on the defined “treated hemiface” once a day, in the evening, according to a randomization list. Subjects were required to complete the daily log and return the used test product at the end of the study.

The hydrating cream was applied from D8 to M3 in the morning to the entire face and in the evening, to the control hemiface alone.

### 2.4 Outcomes & Assessment of Physical and Functional Dermatological Signs

The primary outcome was the dermatological tolerance of the test product in all subjects after 3 months of once‐daily use on the hemiface.

The secondary efficacy outcomes compared the treated area versus the control area and included the physician's clinical scoring of the severity of crow's feet wrinkles, and the firmness, smoothness and plumpness of the skin, as well as the radiance of complexion, skin texture, and homogeneity of tone evaluated by the technicians using a clinical score based on photographs at M1, M2 and M3.

The visits were performed at D1, D8, D8 + 1 month (D37, M1), D8 + 2 months (D66, M2), and D8 + 3 months (D95, M3). A window of ±24 h was allowed for M1 and M2 and +72 h for M3 for subjects continuing test product application until the day before the final visit.

All clinical evaluations were performed by a single dermatologist at D8 (before and immediately after product application, D8Timm), M1, M2, and M3. Assessments included physical and functional signs rated on a 5‐point numerical rating scale. Any new or worsened reactions were documented. Global tolerance was graded by the investigator as excellent, very good, good, moderate or poor.

Clinical scoring of aging signs was performed by an investigator. The severity of crow's feet wrinkles was assessed using the Bazin's score, ranging from 0 (no wrinkles) to 6 (very deep wrinkles) [15]. Firmness, smoothness, and plumpness were scored from 0 (lack of firmness, smoothness, and plumpness) to 10 (firm, smooth and plump skin). The percentage numbers of subjects showing improvement, no change or worsening were determined.

Macro photographs were taken by the technician using a Nikon lens in standard light at D8 (before application of the test product), M1, M2 and M3 for clinical scoring. The device used to take the illustrative photographs was a Visia module (Canfield Imaging Systems, USA), which rotates around the subject's face and takes left, right and central facial views to produce high‐resolution, standardized facial images.

The radiance of the complexion was scored by the technicians on a 10‐point scale from 0 (dull complexion) to 10 (radiant complexion) using the photographs. Skin texture was also assessed on a 10‐point score from 0 (rough) to 10 (fine) using the photographs, as was homogeneity of tone, with scores ranging from 0 (highly uneven) to 10 (even tone).

### 2.5 Statistical Analysis

Statistical analysis was performed using SAS 9.4 (Institute Inc., NC, USA).

Tolerance analyses were performed on the full analysis set (FAS) comprising all subjects who applied the test product at least once.

Change over baseline (intragroup) and comparison between treated and control areas (intergroup) were calculated for the clinical NRS scores and for the percentages of subjects exhibiting improvement, no change or worsening.

Intragroup and intergroup comparisons were performed using the paired *t*‐test, or the Wilcoxon signed rank test where the outcome did not follow a Gaussian distribution.

Statistical significance was set at 5% for all comparisons.

## 3 Results

### 3.1 Participants

Overall, 66 subjects (58 women and 8 men) aged from 35 to 69 years [mean: 47 years] were included (Table 1) and underwent fractional ablative laser (*N* = 22), peeling with 30% or 50% glycolic acid (*N* = 22) or injections with HA, or a mixture comprising HA (*N* = 22) (Figure S1). Thirty‐five percent of participants declared having sensitive skin. Two subjects discontinued the study due to signs of skin irritation (erythema, skin dryness and sensations of tightness and burning): one on M1 (Day 37) following persisting moderate reactions related to the test product, and the other on Day 14, related to the hydrating cream. In both cases, regenerating preparations were recommended at the time of withdrawal, and symptoms resolved by Day 19 for the latter.

**TABLE 1:** Baseline demographic and clinical characteristics of the study population.

Parameters at baseline (*n* = 66)	
Age (years) mean ± SEM	49 ± 1
Median [min–max]	47 [35–69]
Gender, *n*, female/male	58/8
Skin phototype, *n* (%)	I: 2 (3%) II: 61 (92%) III: 3 (5%)
Facial skin type, *n* (%)	Normal: 46 (70%) Dry: 1 (1%) Oily: 4 (6%) Combination: 15 (23%)
Contact lenses wearer	Yes: 8 (12%) No: 58 (88%)
Cosmetic procedure	Injection: 22 (33%) Laser: 22 (33%) Peeling: 22 (33%)
Sensitive skin	Yes: 23 (35%) No: 43 (65%)
Number of subjects withdrawn from study	2*
Number of subjects in analysis of results	D8: 65 subjects M1: 62 subjects M2: 63 subjects M3: 59 subjects

Abbreviation: SEM, standard error of the mean.

* Due to intolerance reaction: 1 subject at M1 (irritation related to the test product) and 1 subject before M1 (irritation related to the hydrating cream).

Compliance was high and most subjects adhered to the daily regimen, with only minor deviations in application frequency by a small number of patients over 3 months.

### 3.2 Dermatological Tolerance and Safety (Primary Criterion)

Ten subjects reported reactions likely related to the study product, and these occurred during the first month for 8 of the 10 (Table S1). Four subjects (6%) reported only functional signs (e.g., stinging, burning, or tightness) that were mostly ephemeral and mild and occurred only on D8Timm, while 4 (6%) reported only physical signs (such as mild transient erythema), and 6 (9%) reported both functional and physical signs.

No serious AEs occurred during the study period. The test product was judged by the dermatologist as having a moderate dermatological tolerance profile during the post‐procedure period.

### 3.3 Changes in Clinical Aging

#### 3.3.1 Reduction of Wrinkles

**FIGURE 1:**
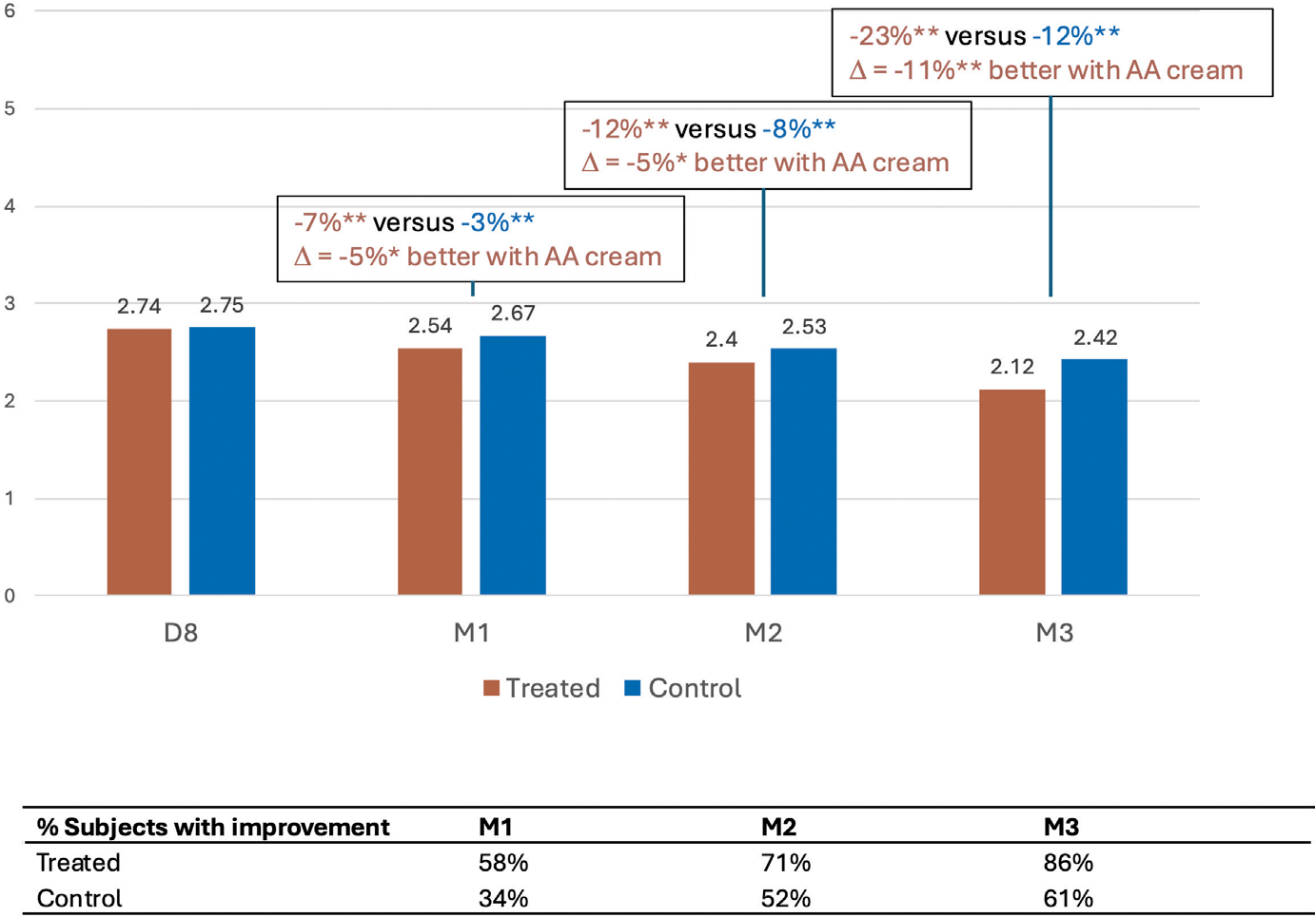
Reduction of crow's feet wrinkles with the anti‐aging (AA) cream.

Investigator‐assessed crow's feet wrinkles showed progressive improvement from M1 onward, with significantly greater reduction in the treated area compared to the control area (Figure 1). Severity, measured using Bazin's score, decreased very significantly in the treated area by −7%, −12%, and −23% at M1, M2, and M3, respectively, versus −3%, −8%, and −12% in the control area. The differences between the treated and control hemiface at each time point were 5%, 5%, and 11%, demonstrating a significantly greater reduction in wrinkle severity with the study cream. Additionally, the percentage of subjects showing clinical improvement increased consistently across all visits.

#### 3.3.2 Firmness, Plumpness and Smoothness of the Skin

**FIGURE 2:**
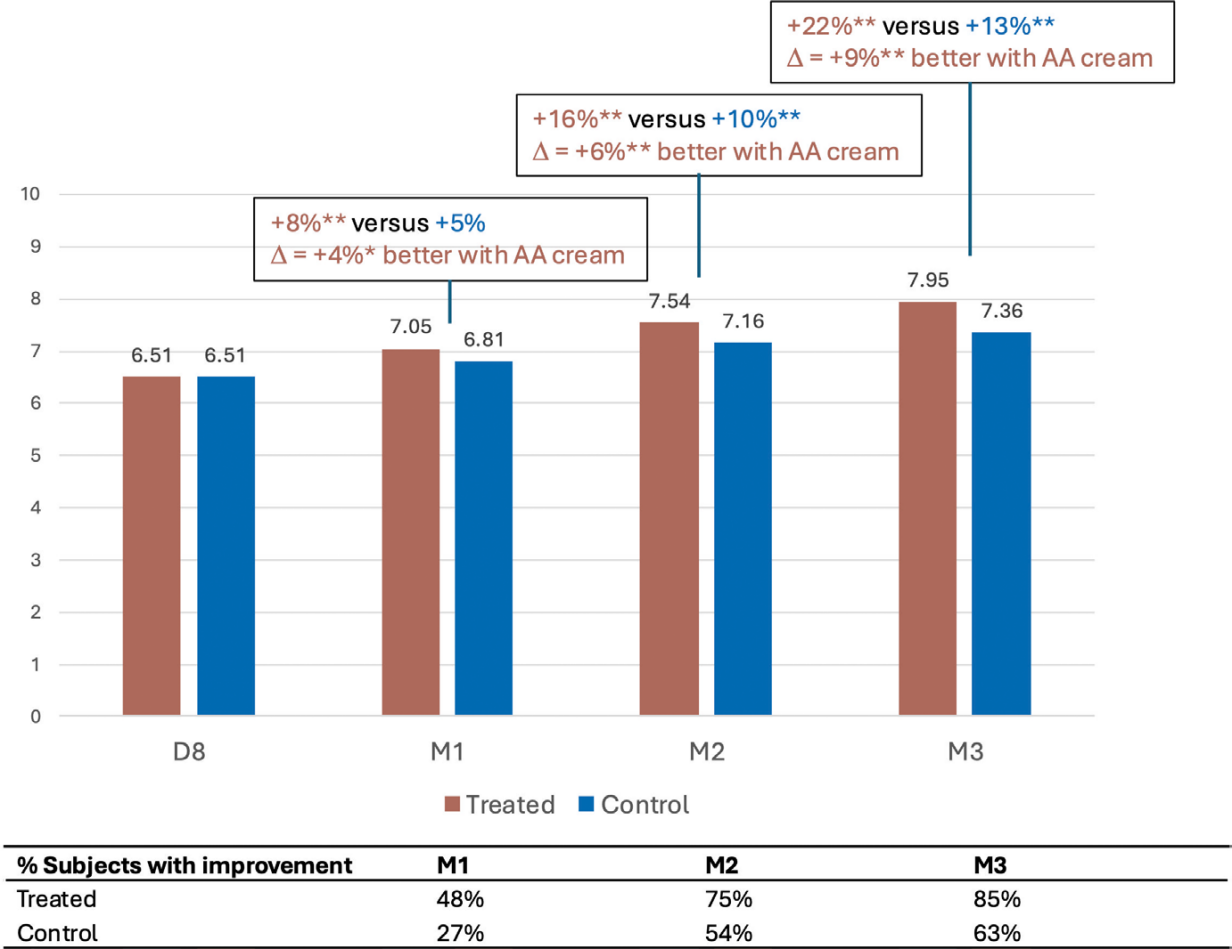
Increase in skin firmness with the anti‐aging (AA) cream.

Following the three types of procedure, investigator‐assessed firmness of the skin improved at all visits from M1 onwards. Greater improvement was seen in the treated area, as attested by the significant increase in clinical score by +8%, +16%, and +22% at M1, M2, and M3, respectively, versus +5%, +10%, and +13% in the control area, as well as by the percentage number of subjects exhibiting improvement at all visits (Figure 2). Skin firmness was significantly higher in the treated area compared with the control area at 4% (*p* = 0.0012) at M1, 6% (*p* < 0.0001), at M2, and 9% (*p* < 0.0001) at M3.

**FIGURE 3:**
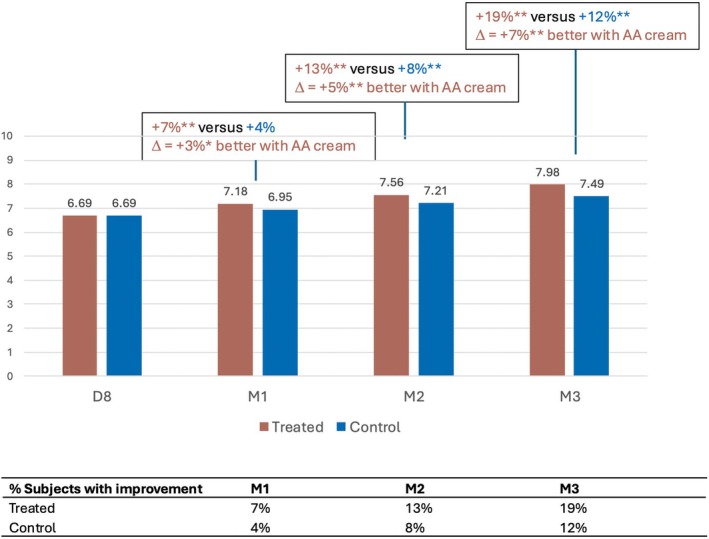
Increase in skin plumpness with the anti‐aging (AA) cream.

**FIGURE 4:**
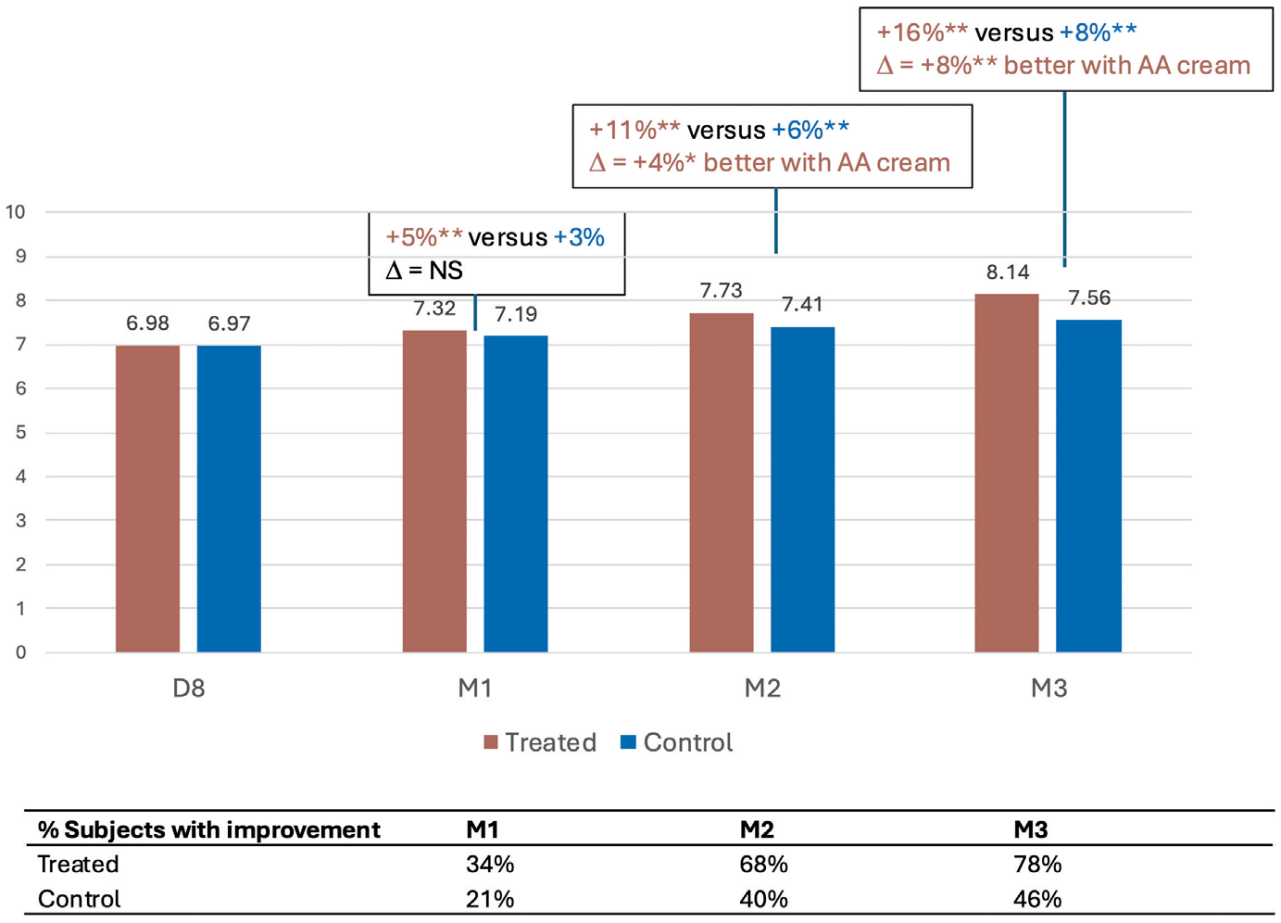
Increase in skin smoothness with the anti‐aging (AA) cream.

Similarly, skin plumpness (Figure 3) and smoothness (Figure 4) were significantly enhanced in the treated area, from M1 onwards and M2 onwards, respectively, as shown by the significantly increased mean NRS grades. The investigator rated skin plumpness as greater by +7%, +13% and +19% in the treated area versus +3%, +5%, and +7% in the control area at M1, M2, and M3 respectively. Similarly, skin smoothness improved progressively by +5%, +11%, and +16% in the treated area, versus +3%, +6%, and +8% in the control area at the same time points.

### 3.4 Quality of the Skin Evaluated on Photographs

**FIGURE 5:**
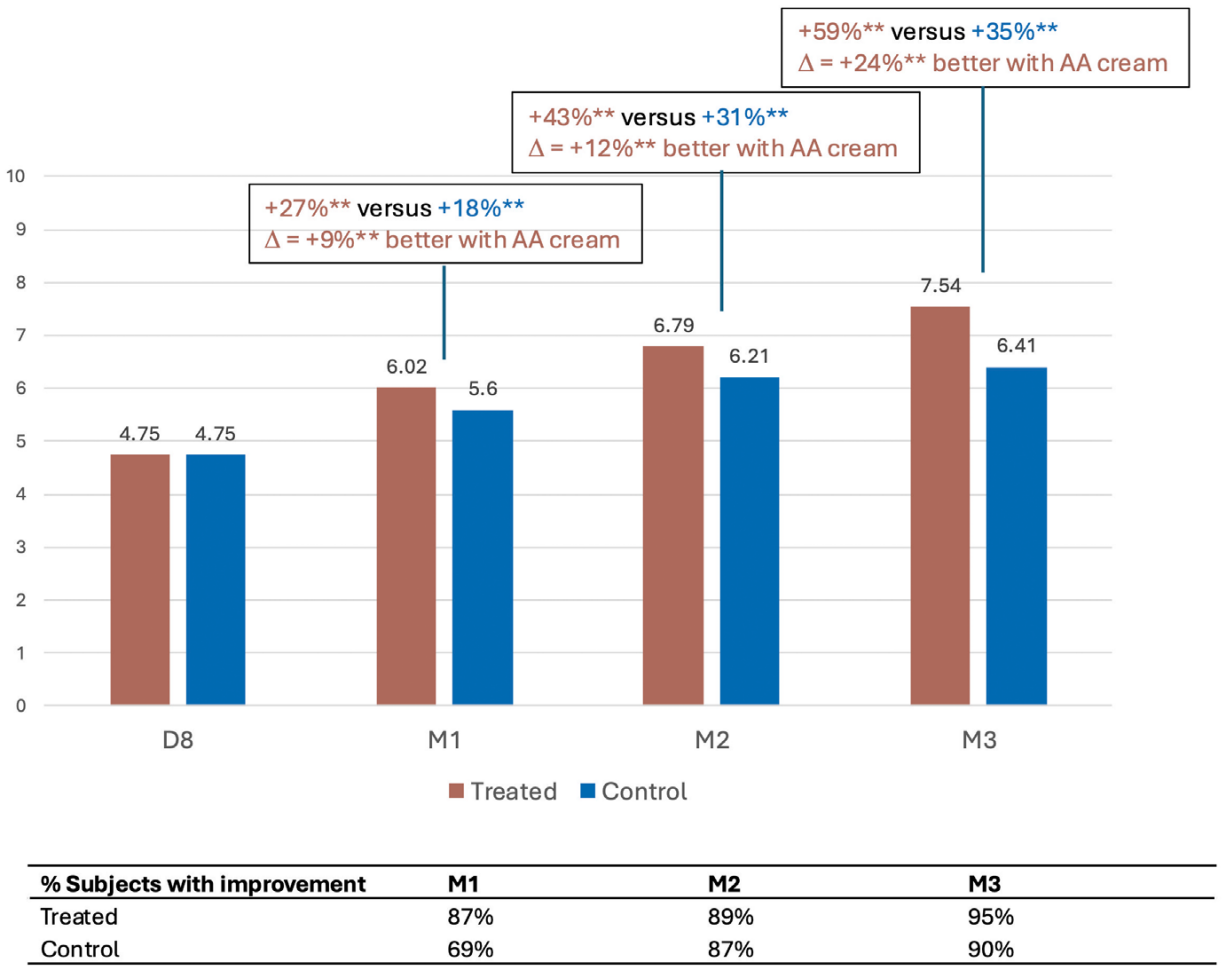
Increase in radiance of complexion with the anti‐aging (AA) cream.

The radiance and brightness of the complexion as assessed by the technicians using a clinical score based on the photographs were all significantly higher in the treated area than in the control area from M1 onwards (Figure 5).

This parameter increased very significantly by +27%, +43%, and +59% in the treated area versus +18%, +31% and +35% in the control area at M1, M2, and M3 respectively. The difference in clinical scores between the treated and control areas at M1, M2, and M3 was 9% (*p* < 0.0001), 12% (*p* < 0.0001), and 24% (*p* < 0.0001), respectively. Moreover, the percentage of improved subjects was very high and even greater in the treated area than in the control area at M1 (87% vs. 69%), M2 (89% vs. 87%), and M3 (95% vs. 90%).

**FIGURE 6:**
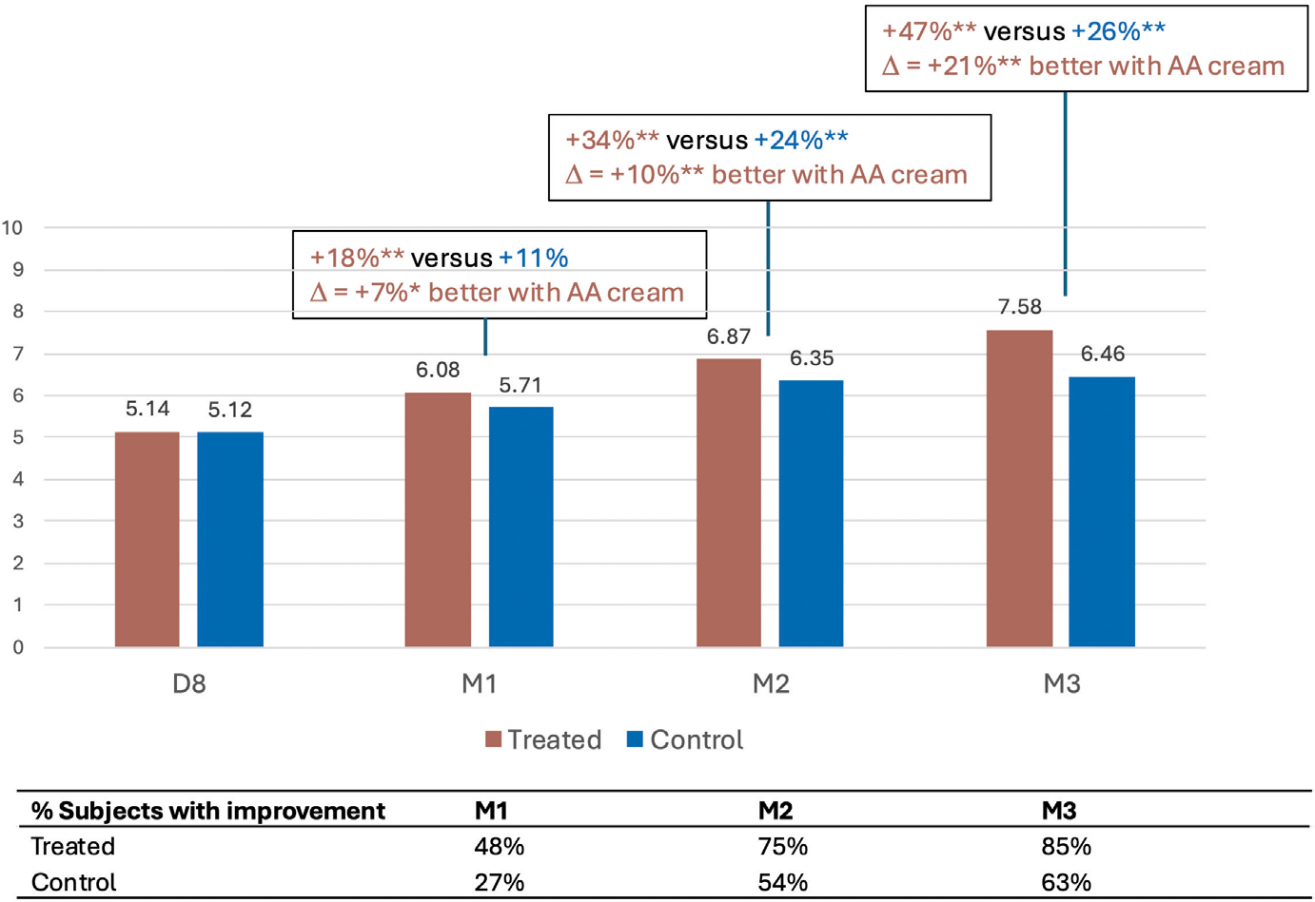
Improvement in skin texture with the anti‐aging (AA) cream.

**FIGURE 7:**
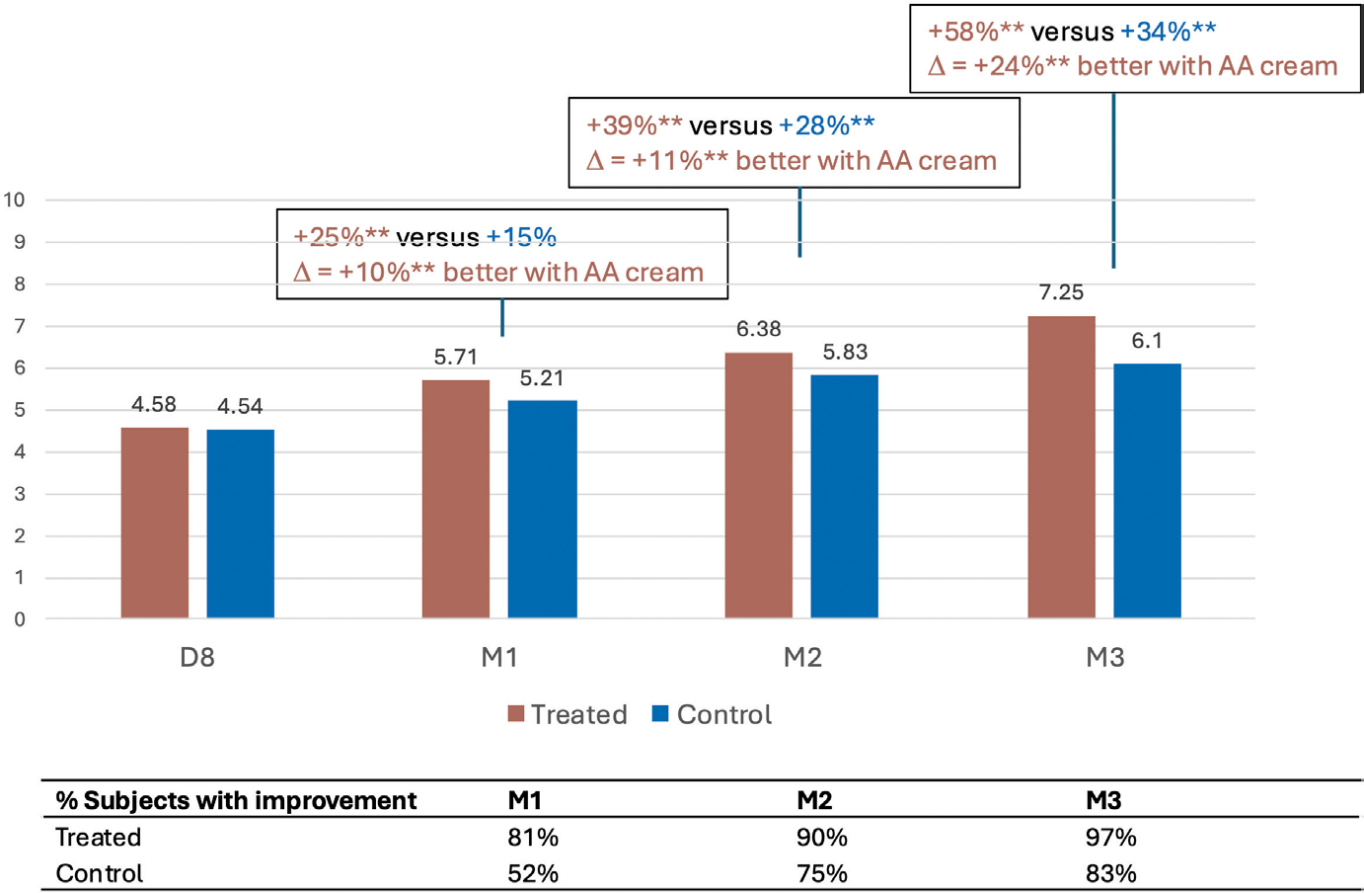
Increase in homogeneity of skin tone with the anti‐aging (AA) cream.

Similarly, skin texture (Figure 6) and homogeneity of tone (Figure 7) were significantly improved in the treated area from M1 onwards. The investigator rated skin as 7%, 10%, and 21% finer compared with the control hemiface, and skin tone as 10%, 11%, and 24% more even compared with the control hemiface at M1, M2, and M3, respectively.

**FIGURE 8:**
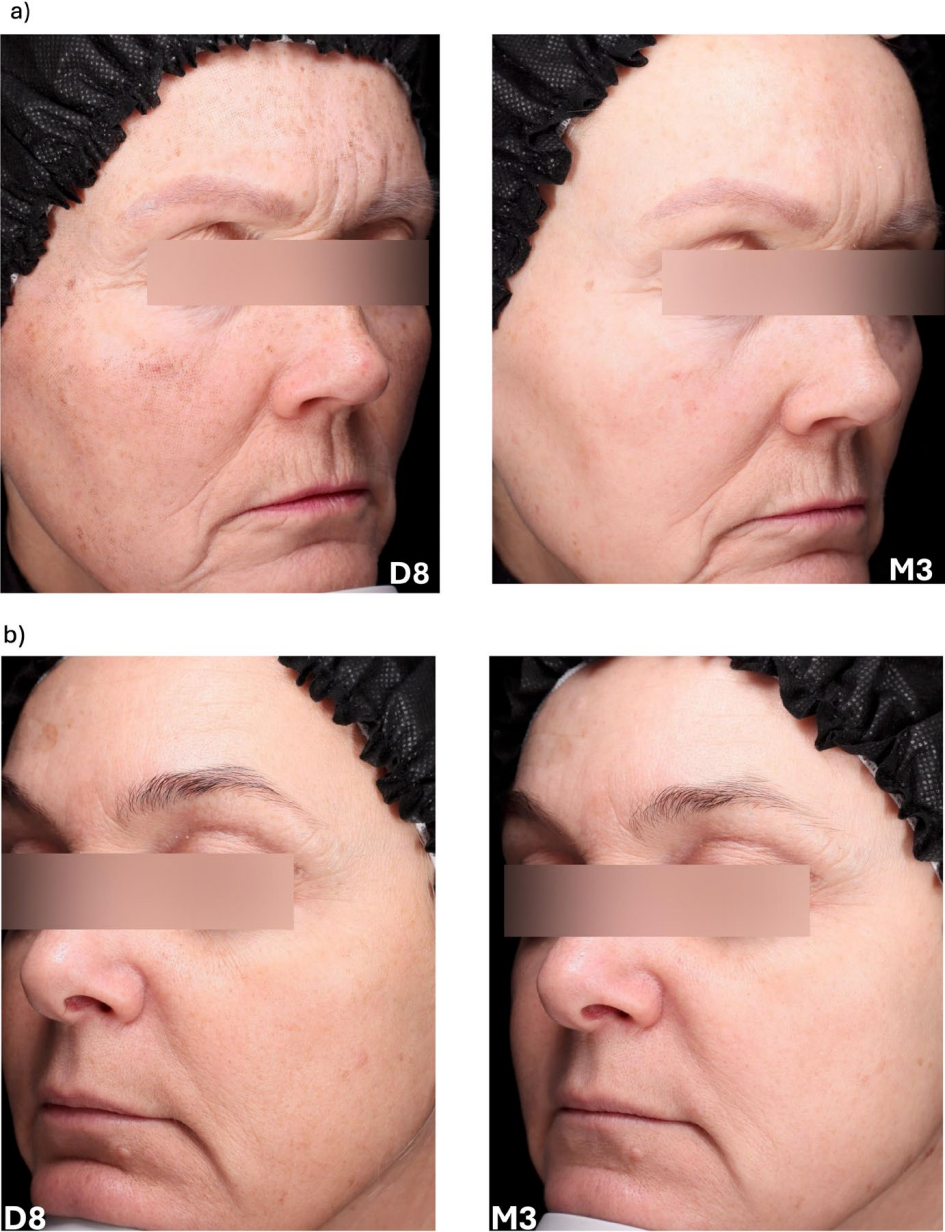
Illustrative clinical photographs at D8 and M3 in subjects undergoing (a) fractional ablative lase, (b) peeling with 30% or 50% glycolic acid.

Overall, all the aging parameters were significantly improved in the treated versus control area from M1, except for skin smoothness, which showed significant improvement from M2. Maintenance and enhancement of the anti‐aging effect of the rejuvenation procedures obtained using the study cream were illustrated by the clinical photographs at D8 and M3 (Figure 8).

## 4 Discussion

This monocentric, randomized split‐face study collected data on the tolerance and efficacy of a cream containing retinaldehyde, niacinamide and a dry extract of haritaki fruit in maintaining and enhancing the anti‐aging results of a minimally to moderately invasive rejuvenation procedure in 66 subjects. This comparative study, conducted under close dermatological supervision with a 3‐month follow‐up, demonstrated that the cream improved multiple signs of aging. Compliance was very good throughout the entire study. After 1 month, wrinkles were significantly reduced, and the skin was significantly firmer and plumper, with improved texture. Skin tone homogeneity and radiance were significantly enhanced from M1 (and from M2 for skin smoothness).

Tolerance was judged as moderate by the dermatologist. This conclusion was based on the usual side effects encountered after undergoing minimally to moderately invasive rejuvenation. Indeed, the temporarily compromised skin barrier increases the risk of sensitivity and irritation. Furthermore, 35% of the participants had sensitive skin at inclusion and were thus more susceptible to adverse reactions to the product, since their skin was inherently more sensitive to irritation [16] or “the occurrence of unpleasant sensations (stinging, burning, pain, pruritus, and tingling sensations) in response to stimuli that normally should not provoke such sensations.” [17].

Only usual and expected local side effects related to the ingredients of the cream were observed. Retinoids, particularly retinoic acid, are known to cause skin irritation, and retinoic acid is restricted to prescription treatments. The test product contains retinaldehyde, which is transformed in small amounts to retinoic acid in the skin, and is the most potent retinoid available in over‐the‐counter products [12]. Retinaldehyde has demonstrated good tolerability, enabling prolonged use for up to 3 years [18, 19, 20], with habituation typically occurring after the first month of use [19]. Likewise, in our study, the local side effects likely or very likely associated with the test product occurred during the first month in 8 of 10 subjects. By contrast, niacinamide is well tolerated, even in individuals with sensitive skin conditions like rosacea [21].

The anti‐aging cream has undergone multiple tolerance studies outside the post‐procedure context, demonstrating good skin tolerance and excellent ocular tolerance. In the absence of established clinical practice guidelines, a stepwise approach was followed for the development of cosmetic skincare products [22]. In vitro assessments preceded clinical evaluations, including consumer testing, Human Repeated Insult Patch Test (HRIPT), phototoxicity, dermatological and ocular tolerance studies, biometrology efficacy analysis, and a comedogenicity study. Where the anti‐aging cream is used as a post‐procedure treatment, dermatologists should balance potency with tolerability for each individual patient, if necessary, delaying the introduction of the cream to ensure optimal patient compliance.

Minimally to moderately rejuvenation procedures can achieve even better results when combined with cosmeceuticals as post‐procedure adjuvants [10]. When used after three types of minimally‐to‐moderately invasive rejuvenation procedures—two involving cutaneous resurfacing and one using a filler—the cream proved effective in maintaining and enhancing anti‐aging outcomes. Use of the split‐face study design ensures reliable results after 3 months of once‐daily application. The anti‐aging effect of the product was assessed globally rather than separately for each of the three procedure types, since each targets specific concerns and our aim was to evaluate overall anti‐aging efficacy without isolating individual contributions. The cream was shown to target multiple signs of aging, with significant reduction of wrinkle depth, and improvement of both skin texture (more fine, smooth and plump) and complexion (more radiant and even) on the treated hemiface compared to the control. Of note is the fact that these effects were discernible from M1 (and from M2 for smoothness).

These multiple effects on signs of skin aging can be accounted for by the combination of highly concentrated active ingredients [12]. Retinoids inhibit collagen degradation, promote keratinocyte proliferation, collagen synthesis, and induction of hyaluronan production [20, 23, 24]. Niacinamide, a derivative of vitamin B3, improves skin pigmentation and skin surface irregularity [25, 26, 27]. Haritaki fruit extract provides long‐lasting antioxidant protection by effectively neutralizing reactive oxygen species (ROS) and reducing inflammation by inhibiting cytokines such as IL‐6 and IL‐8. This helps reverse pollution‐induced skin damage and improves skin texture, hydration, and firmness [13]. Furthermore, one cause of functional decline of skin with age is the accumulation of senescent cells, which release detrimental molecules and exosomes on exiting from the cell cycle. Dry extract of haritaki fruit was shown to inhibit the deleterious effects of senescent cells by reducing SASP (“senescence‐associated secretory phenotype”) gene expression and microRNAs in senescent fibroblasts [14]. Together, these active ingredients exert complementary activities on the various mechanisms of skin aging that enable the study cream to improve facial appearance globally.

In our study, the subjective evaluation of the participants was consistent with the investigator's clinical assessment, and after 84 days of use, over 90% of subjects reported a satisfaction score of 5 or higher (on a 10‐point NRS) regarding the appearance of their skin and the maintenance and enhancement of the rejuvenation procedure (Table S2).

We acknowledge that our controlled study included mostly women, of phototypes I–III, which potentially limits its generalizability. In particular, it might be of interest to assess the efficacy of the anti‐aging cream on post‐inflammatory hyperpigmentation, which is a common concern in people with higher skin phototypes [28, 29]. Additionally, we did not test our anti‐aging skincare product following all the various types of aesthetic procedures available, but only the three main types of minimally to moderately invasive procedures. Interestingly, the fact that this product was tolerated even after CO_2_ fractional treatment suggests that it would likely be tolerated for many, if not all, less aggressive cosmetic procedures. According to a very recent international Delphi consensus study, topical retinoids could be applied as early as 1 day after a non‐energy, non‐ablative laser procedure [30]. However, the laser used in our study was ablative, affecting the epidermis. In the future, the test product could be applied at an earlier stage following the rejuvenation procedure, for example after an HA injection, to meet patient needs.

However, this study demonstrated good results with a robust design in 66 subjects. Moreover, a within‐person design reduces interindividual variability, while enhancing statistical power and allowing fewer participants compared to parallel‐group studies, where each receives only one intervention. In addition, compliance was very good, despite the constraints of hemiface application over a 3‐month period.

In conclusion, the incorporation of dermocosmetics into a skincare routine can improve the results of dermatological anti‐aging procedures by maintaining and prolonging the effects of treatments. However, it is essential to choose carefully formulated products and consult a dermatologist for personalized recommendations.

The results of the study confirm that the anti‐aging effects of the three types of facial rejuvenation procedures undergone by the 66 participants—peeling, fractional ablative laser, and injections of hyaluronic acid in the crow's foot area—were maintained and enhanced by the study product, which contained retinaldehyde, niacinamide, and haritaki fruit extract, with visible results being noted as early as 1 month coupled with very good skin tolerance. Regular use of these active ingredients in dermocosmetic products helps to maximize the benefits of rejuvenation procedures, maintain their effects, and promote long‐term skin health.

## Author Contributions

All named authors meet the International Committee of Medical Journal Editors (ICMJE) criteria for authorship of this article, take responsibility for the integrity of the work as a whole, and have provided their approval for publication of this version. C.M., T.N., E.G., and G.D. participated in the conception and design of the study. C.M. and T.N. contributed to the data analysis. The draft of the manuscript was reviewed and commented on by all authors. All authors have read and approved the final manuscript.

## Ethics Statement

This study was performed in accordance with the principles stated in the Declaration of Helsinki and subsequent amendments (1964, and most recent amendment in force), and in accordance with the spirit of Good Clinical Practice (GCP) Guideline published by the International Conference on Harmonization (Adopted by CHMP on December 15, 2016, issued as EMA/CHMP/ICH/135/1995) and with local regulatory requirements. The protocol was approved by a local ethics committee. It also conformed to Regulation (EC) No. 1223/2009 of the European Commission and the French Decree (No. 2017‐884) pertaining to research on cosmetic products.

## Consent

Informed consent (and an authorization photograph) was obtained from all subjects during inclusion.

## Conflicts of Interest

V.B.‐C. and E.L. have received honoraria for presentations and for participation in expert meetings from Pierre Fabre. C.M., E.G., G.D., and T.N. are employed by Pierre Fabre.

## Supporting information


**Figure S1:** Study flow chart.

**Table S1:** Dermatological tolerance and safety.
**Table S2:** Subjective evaluation by patients: quantitative answers to a questionnaire on a scale of 1 to 10.

## Data Availability

The data that support the findings of this study are available on request from the corresponding author. The data are not publicly available due to privacy or ethical restrictions.

## References

[1] J. M. Amici , O. Cogrel , M. Jourdan , et al., “Expert Recommendations on Supportive Skin Care for Non‐Surgical and Surgical Procedures,” Journal of the European Academy of Dermatology and Venereology 37 (2023): 16–33, 10.1111/jdv.18855. 36635618

[2] S. Meghe , R. Ramapure , S. Jaiswal , S. Jawade , S. Singh , and S. R. Meghe , “A Comprehensive Review of Minimally Invasive Dermatosurgical Procedures,” Cureus 16 (2024): e56152. 38618325 10.7759/cureus.56152 PMC11015872

[3] J. Sharad , “Glycolic Acid Peel Therapy—A Current Review,” Clinical, Cosmetic and Investigational Dermatology 6 (2013): 281–288, 10.2147/ccid.s34029. 24399880 PMC3875240

[4] K. Beasley , M. Weiss , and R. Weiss , “Hyaluronic Acid Fillers: A Comprehensive Review,” Facial Plastic Surgery 25 (2009): 86–94, 10.1055/s-0029-1220647. 19415575

[5] E. P. Tierney , R. F. Eisen , and C. W. Hanke , “Fractionated CO_2_ Laser Skin Rejuvenation,” Dermatologic Therapy 24 (2011): 41–53, 10.1111/j.1529-8019.2010.01377.x. 21276157

[6] S. S. Collawn , “Re‐Epithelialization of the Skin Following CO_2_ Laser Resurfacing,” Journal of Cutaneous Laser Therapy 3 (2001): 123–127, 10.1080/147641701753414924. 12006188

[7] R. J. Hirsch , S. H. Dayan , and A. R. Shah , “Superficial Skin Resurfacing,” Facial Plastic Surgery Clinics of North America 12 (2004): 311–321, 10.1016/j.fsc.2004.02.006. 15261168

[8] J. D. Wisniewski , D. L. Ellis , and M. P. Lupo , “Facial Rejuvenation: Combining Cosmeceuticals With Cosmetic Procedures,” Cutis 94 (2014): 122–126. 25279473

[9] M. Lupo and L. Jacob , Cosmeceuticals for Enhancing Cosmetic Procedures (John Wiley & Sons, Ltd, 2013), 268–276.

[10] K. Y. Park and I. L. Gehrke , “Combined Multilevel Anti‐Aging Strategies and Practical Applications of Dermocosmetics in Aesthetic Procedures,” Journal of the European Academy of Dermatology and Venereology 38 (2024): 23–35, 10.1111/jdv.19975. 38881448

[11] W. Barańska‐Rybak and M. Antoszewska , “Combination of Hyaluronic Acid Fillers and Personalized Skincare as a Perfect Tool in Aesthetic Medicine,” Dermatologic Therapy 34 (2021): 15092, 10.1111/dth.15092. 34369044

[12] Z. Draelos , P. Bogdanowicz , and J. H. Saurat , “Top Weapons in Skin Aging and Actives to Target the Consequences of Skin Cell Senescence,” Journal of the European Academy of Dermatology and Venereology 38 (2024): 15–22, 10.1111/jdv.19648. 38881445

[13] M. Randhawa , T. Meyer , M. Sachdev , and R. K. Chaudhuri , “Standardized *Terminalia chebula* Fruit Extract: A Natural Ingredient That Provides Long‐Lasting Antioxidant Protection and Reverses Visible Signs of Pollution‐Induced Skin Damage,” Clinical, Cosmetic and Investigational Dermatology 14 (2021): 1257–1269, 10.2147/ccid.s326492. 34557011 PMC8456126

[14] P. Bogdanowicz , N. Roullet , P. Bensadoun , S. Bessou‐Touya , J. M. Lemaitre , and H. Duplan , “Reduction of Senescence‐Associated Secretory Phenotype and Exosome‐Shuttled miRNAs by Haritaki Fruit Extract in Senescent Dermal Fibroblasts,” International Journal of Cosmetic Science 45 (2023): 488–499, 10.1111/ics.12858. 36940283

[15] R. Bazin and E. Doublet , “Skin Aging Atlas,” in Caucasian Type, vol. 1 (MED'COM, 2007).

[16] H. I. Maibach , K. Lammintausta , E. Berardesca , and S. Freeman , “Tendency to Irritation: Sensitive Skin,” Journal of the American Academy of Dermatology 21 (1989): 833–835. 2689477 10.1016/s0190-9622(89)70259-0

[17] L. Misery , S. Ständer , J. Szepietowski , et al., “Definition of Sensitive Skin: An Expert Position Paper From the Special Interest Group on Sensitive Skin of the International Forum for the Study of Itch,” Acta Dermato‐Venereologica 97 (2017): 4–6, 10.2340/00015555-2397. 26939643

[18] J. H. Saurat , L. Didierjean , E. Masgrau , et al., “Topical Retinaldehyde on Human Skin: Biologic Effects and Tolerance,” Journal of Investigative Dermatology 103 (1994): 770–774, 10.1111/1523-1747.ep12412861. 7798613

[19] J. W. Fluhr , M. P. Vienne , C. Lauze , P. Dupuy , W. Gehring , and M. Gloor , “Tolerance Profile of Retinol, Retinaldehyde and Retinoic Acid Under Maximized and Long‐Term Clinical Conditions,” Dermatology 199 (1999): 57–60, 10.1159/000051381. 10473963

[20] J. E. Kim , B. Kim , H. Kim , et al., “Retinyl Retinoate Induces Hyaluronan Production and Less Irritation Than Other Retinoids,” Journal of Dermatology 37 (2010): 448–454, 10.1111/j.1346-8138.2010.00808.x. 20536650

[21] Z. D. Draelos , K. Ertel , and C. Berge , “Niacinamide‐Containing Facial Moisturizer Improves Skin Barrier and Benefits Subjects With Rosacea,” Cutis‐New York 76 (2005): 135. 16209160

[22] V. Ribet , M. Gurdak , P. J. Ferret , E. Brinio , F. Giordano Labadie , and A. B. Rossi , “Stepwise Approach of Development of Dermo‐Cosmetic Products in Healthy and Atopic Dermatitis Paediatric Population: Safety Evaluation, Clinical Development and Postmarket Surveillance,” Journal of the European Academy of Dermatology and Venereology 33 (2019): 2319–2326, 10.1111/jdv.15785. 31287596 PMC6900091

[23] S. Mukherjee , A. Date , V. Patravale , H. C. Korting , A. Roeder , and G. Weindl , “Retinoids in the Treatment of Skin Aging: An Overview of Clinical Efficacy and Safety,” Clinical Interventions in Aging 1 (2006): 327–348, 10.2147/ciia.2006.1.4.327. 18046911 PMC2699641

[24] S. Kang , “The Mechanism of Action of Topical Retinoids,” Cutis 75 (2005): 10–13. 15773538

[25] Z. D. Draelos , “The Latest Cosmeceutical Approaches for Anti‐Aging,” Journal of Cosmetic Dermatology 6 (2007): 2–6, 10.1111/j.1473-2165.2007.00313.x. 17524121

[26] D. L. Bissett , J. E. Oblong , and C. A. Berge , “Niacinamide: AB Vitamin That Improves Aging Facial Skin Appearance,” Dermatologic Surgery 31 (2005): 860–866. 10.1111/j.1524-4725.2005.31732 16029679

[27] Y. C. Boo , “Mechanistic Basis and Clinical Evidence for the Applications of Nicotinamide (Niacinamide) to Control Skin Aging and Pigmentation,” Antioxidants 10 (2021): 1315. 34439563 10.3390/antiox10081315 PMC8389214

[28] P. Adotama , N. Papac , A. Alexis , A. Wysong , and L. Collins , “Common Dermatologic Procedures and the Associated Complications Unique to Skin of Color,” Dermatologic Surgery 47 (2021): 355–359, 10.1097/dss.0000000000002813. 34328287

[29] I. T. Y. Wong and V. Richer , “Prophylaxis of Post‐Inflammatory Hyperpigmentation From Energy‐Based Device Treatments: A Review,” Journal of Cutaneous Medicine and Surgery 25 (2021): 77–86, 10.1177/1203475420957633. 32929988

[30] A. F. Alexis , A. Andriessen , R. A. Beach , et al., “Periprocedural Skincare for Nonenergy and Nonablative Energy‐Based Aesthetic Procedures in Patients With Skin of Color,” Journal of Cosmetic Dermatology 24 (2025): e16712, 10.1111/jocd.16712. 39829119 PMC11744056

