# Supplementary material for: Enhancing Facial Rejuvenation Outcomes With a Novel Retinaldehyde‐Based Cream: A Comparative Randomized Intra‐Individual Study
Source: J Cosmet Dermatol. 2025 Nov 28;24(12):e70555. doi: 10.1111/jocd.70555 (PMC12661472; doi:10.1111/jocd.70555)
Supplement: Supplementary file 1 — Figure S1: Study flow chart. [file JOCD-24-e70555-s001.docx]

Analyzed

Full analysis set:

- *N = 22 at D8,*
- *N = 21 at D37*
- *N = 20 at D66, D95*

Per protocol analysis set:

- *N = 21 at D8,*
- *N = 20 at D37, D66*
- *N = 19 at D95*

Analyzed

Full analysis set: N = 22

Per protocol analysis set:

- *N = 22 at D8*
- *N = 21 at D37, D66, D95*

Analyzed

Full analysis set: N = 22

Per protocol analysis set:

- *N = 22 at D8, D66*
- *N = 21 at D37,*
- *N = 19 at D95*

Allocated to the laser group

*N = 22*

Allocated to the injection group

*N = 22*

Prematurely withdrawn

- Intolerance reaction (*n* = 2)

Allocated to the peeling group

*N = 22*

Included

*N = 66*
