# Supplementary material for: Enhancing Facial Rejuvenation Outcomes With a Novel Retinaldehyde‐Based Cream: A Comparative Randomized Intra‐Individual Study
Source: J Cosmet Dermatol. 2025 Nov 28;24(12):e70555. doi: 10.1111/jocd.70555 (PMC12661472; doi:10.1111/jocd.70555)
Supplement: Supplementary file 2 — Table S1: Dermatological tolerance and safety. Table S2: Subjective evaluation by patients: quantitative answers to a questionnaire on a scale of 1 to 10. [file JOCD-24-e70555-s002.docx]

**Supp. Table 1: Dermatological tolerance and safety**

| Reactions related to the one of the study products | N | % |
| --- | --- | --- |
| **Total of subjects presenting reactions** | **14** | **21** |
| Subjects exhibiting functional signs only | 4 | 6 |
| Subjects exhibiting physical signs only | ~~4~~ | 6 |
| Subjects exhibiting both functional and physical signs | 6 | 9 |
| Subjects exhibiting reactions likely or very likely related to the test product | 10 | 15 |
| Subjects with SAE related to the test product | 0 | 0 |
| Subjects with reactions observed by the investigator | 7 | 11 |
| Subjects who have modified modalities of application and/or temporarily interrupted the product because of the reaction | 1 | 2 |

**Supp. Table 2:** Subjective evaluation by patients: quantitative answers to a questionnaire on a scale of 1 to 10

| Questionnaire | Mean value on a scale of 1 - 10 | Number of subjects with a score ≥ 5 | Percentage of subjects with a score ≥ 5 |
| --- | --- | --- | --- |
| **“After 84 days of use, you would say that your skin is (on the treated side vs. non treated side)”:** |  |  |  |
| Firmer | 8.2 | 36 | 95% |
| More radiant | 8.1 | 36 | 95% |
| Less wrinkled | 7.9 | 34 | 89% |
| More toned | 7.7 | 35 | 92% |
| Smoother | 8.4 | 35 | 92% |
| Refined skin texture | 8.0 | 35 | 92% |
| **“After 84 days of use, you would say that the product”:** |  |  |  |
| Helps maintain the effects of the dermatological procedure | 8.4 | 36 | 95% |
| Completes the results of the dermatological procedure | 8.3 | 35 | 92% |
| Boosts the results of the dermatological procedure | 8.3 | 36 | 95% |
